# Comparison of gene expression of *Paramecium bursaria* with and without *Chlorella variabilis* symbionts

**DOI:** 10.1186/1471-2164-15-183

**Published:** 2014-03-10

**Authors:** Yuuki Kodama, Haruo Suzuki, Hideo Dohra, Manabu Sugii, Tatsuya Kitazume, Katsushi Yamaguchi, Shuji Shigenobu, Masahiro Fujishima

**Affiliations:** Department of Biological Science, Faculty of Life and Environmental Science, Shimane University, 1060 Nishikawatsu, Matsue, 690-8504 Japan; Department of Environmental Science and Engineering, Graduate School of Science and Engineering, Yamaguchi University, 1677-1 Yoshida, Yamaguchi, 753-8512 Japan; Instrumental Research Support Office, Research Institute of Green Science and Technology, Shizuoka University, 836 Ohya, Suruga-ku, Shizuoka, 422-8529 Japan; Media and Information Technology Center, Yamaguchi University, 2-16-1 Tokiwadai, Ube, 755-8611 Japan; National Institute for Basic Biology, Nishigonaka 38, Myodaiji, Okazaki 444-8585 Japan; Department of Basic Biology, School of Life Science, Graduate University for Advanced Studies, Okazaki, 444-8585 Japan

**Keywords:** *Paramecium bursaria*, *Chlorella variabilis*, Secondary symbiosis, Transcriptome analysis

## Abstract

**Background:**

The ciliate *Paramecium bursaria* harbors several hundred cells of the green-alga *Chlorella* sp. in their cytoplasm. Irrespective of the mutual relation between *P. bursaria* and the symbiotic algae, both cells retain the ability to grow without the partner. They can easily reestablish endosymbiosis when put in contact with each other. Consequently, *P. bursaria* is an excellent model for studying cell–cell interaction and the evolution of eukaryotic cells through secondary endosymbiosis between different protists. Despite the importance of this organism, no genomic resources have been identified for *P. bursaria* to date. This investigation compared gene expressions through RNA-Seq analysis and *de novo* transcriptome assembly of symbiont-free and symbiont-bearing host cells.

**Results:**

To expedite the process of gene discovery related to the endosymbiosis, we have undertaken Illumina deep sequencing of mRNAs prepared from symbiont-bearing and symbiont-free *P. bursaria* cells. We assembled the reads *de novo* to build the transcriptome. Sequencing using Illumina HiSeq2000 platform yielded 232.3 million paired-end sequence reads. Clean reads filtered from the raw reads were assembled into 68,175 contig sequences. Of these, 10,557 representative sequences were retained after removing *Chlorella* sequences and lowly expressed sequences. Nearly 90% of these transcript sequences were annotated by similarity search against protein databases. We identified differentially expressed genes in the symbiont-bearing *P. bursaria* cells relative to the symbiont-free cells, including heat shock 70 kDa protein and glutathione S-transferase.

**Conclusions:**

This is the first reported comprehensive sequence resource of *Paramecium* – *Chlorella* endosymbiosis. Results provide some keys for the elucidation of secondary endosymbiosis in *P. bursaria.* We identified *P. bursaria* genes that are differentially expressed in symbiont-bearing and symbiont-free conditions.

**Electronic supplementary material:**

The online version of this article (doi:10.1186/1471-2164-15-183) contains supplementary material, which is available to authorized users.

## Background

As demonstrated by the evolution of mitochondria and chloroplasts, endosymbiosis is a major driving force behind eukaryotic cell evolution leading to acquisition of new intracellular components and cell diversity [1, 2]. Although endosymbiosis is an important and widespread phenomenon, the mechanisms controlling the establishment of endosymbiosis between different eukaryotic cells are not well understood. In fact, *P. bursaria* cells harbor about 700 symbiotic algae in their cytoplasm [3]. Each alga is enclosed in a perialgal vacuole (PV) membrane derived from the host digestive vacuole (DV) membrane, which protects the alga from the host’s lysosomal fusion [4–6]. Irrespective of the mutual relations between *P. bursaria* and symbiotic algae [7–11], the symbiont-free cells and the symbiotic algae retain the ability to grow without a partner. Symbiont-free cells can be prepared by various means: cultivation under constant dark conditions [12–14], treatment with cycloheximide [3, 15, 16], and treatment with the photosynthesis inhibitor dichlorophenyl dimethylurea (DCMU) [17]. However, symbiotic algae can be isolated by homogenization or by sonication or by the treatment of symbiotic cells with detergent. They can grow outside host cells [18]. Symbiont-free cells are easily reinfected with symbiotic algae by mixing the two together. Therefore, *P. bursaria* has been considered an excellent model for studying cell–cell interaction and the evolution of eukaryotic cells through secondary endosymbiosis between different protists [19]. However, neither genomic nor transcriptomic information has been available to elucidate the establishment of endosymbiosis in *P. bursaria* to date. To expedite the process of gene discovery related to the endosymbiosis, we have undertaken Illumina deep sequencing of mRNAs prepared from symbiont-bearing and symbiont-free *P. bursaria* cells in this study. Our data provide a comprehensive sequence resource for the advancement of *P. bursaria* study.

## Results and discussion

### Deep-sequencing and assembly

We constructed three RNA-seq libraries from mRNA of *P. bursaria* harboring symbiotic alga, *Chlorella variabilis*, and three libraries from symbiont-free *P. bursaria*. Sequencing using Illumina HiSeq2000 platform yielded 232.3 million 101-by-101 bp paired-end sequence reads. After trimming the low-quality parts and removing reads of less than 50 bp, 436.9 million reads (42.9 Gb) remained. To obtain a comprehensive sequence set of the *P. bursaria* transcriptome, all the clean reads of symbiont-bearing and symbiont-free libraries were assembled together using the Trinity program [20]. The *de novo* assembly produced 68,175 contigs, clustering into 40,805 subcomponents (i.e. unigenes). We selected the longest transcript as the representative for each cluster. The unigene sizes were 200 bp up to 22,858 bp, with mean length of 904 bp, N50 of 1,832 bp totaling 36,894,860 bp for all unigenes; 9,620 (23.6%) of unigenes were longer than 1,000 bp.

We excluded unigenes derived from the symbiotic *Chlorella* and other contaminants. Of the 68,175 contig sequences, 11,256 were matched to the *C. variabilis* sequences, and were therefore removed. Unigenes lowly expressed with log-counts-per-million (logCPM) < 0 were also discarded because they are likely to be contaminant sequences or poor assembly models. Based on the database search, the small amount of the contaminant sequences appears to be derived from some bacteria such as *Methylobacterium* and *Burkholderiales*, which are likely to be included in the culture media in which we grew *P. bursaria*. These procedures produced *P. bursaria* transcript reference sequences composed of 10,557 unigenes.

### Annotation of the assembled contigs

We performed similarity searches of the 10,557 *P. bursaria* unigenes against the Swiss-Prot and UniRef90 protein sequence databases [21] using BLASTX [22] with the E-value cutoff of 1e-5 and assigned the functional annotations of the most similar protein sequences. Of the 10,557 unigenes, 7,051 (67%) had matches with 4,102 unique records in the Swiss-Prot database; 9,536 (90%) had matches with 8,189 unique records in the UniRef90 database. The species distribution of the BLASTX best hits in the UniRef90 database showed that 8,710 (91.7%) of the 9,502 hits had top matches with sequences from *P. tetraurelia*, followed by *Tetrahymena thermophila* with 153 (1.6%) best BLASTX hits.

We predicted open reading frames (ORFs) from the 10,557 *P. bursaria* unigene sequences using OrfPredictor [23]. Of the 10,557 ORFs, 10,535 were longer than 50 amino acids, 10,134 were longer than 100 amino acids, and 3,425 were longer than 500 amino acids. Although whole genome sequences have been clarified in *P. tetraurelia*[24] and *T. thermophila*[25], endosymbiotic algae including *Chlorella* species have not yet been detected in these ciliates. Therefore, we tried to compare their ORFs length, GC%, and shared gene clusters among these two ciliates and *P. bursaria* to elucidate the genomic features of *P. bursaria* as a potential host cell for the symbiotic algae.

We compared ORFs (>100 amino acids) of *P. bursaria* with those of its close relatives *P. tetraurelia* and *T. thermophila*. The maximum values for lengths of ORFs for *P. bursaria*, *P. tetraurelia*, and *T. thermophila* were, respectively, 19,640, 21,570, and 34,740. The median values for lengths of ORFs for *P. bursaria*, *P. tetraurelia*, and *T. thermophila* were, respectively, 1,161, 1,155, and 1,503 (Additional file 1). The corresponding values for G + C contents (%) of ORFs were 30.1, 29.7, and 27.8 (Additional file 2).

**Figure 1:**
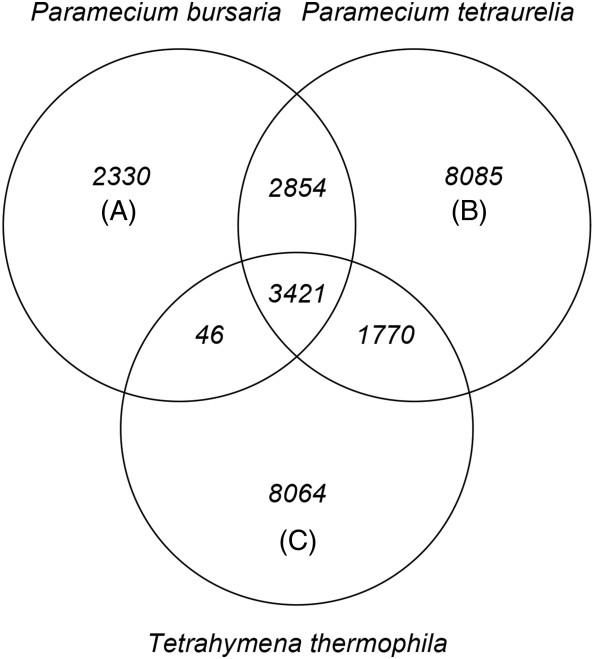
**Venn diagram showing the number of orthologous gene clusters shared among three organisms:** ***P. bursaria*** **(A),** ***P. tetraurelia*** **(B), and** ***T. thermophila*** **(C).** Protein coding sequences from the three organisms were grouped into orthologous gene clusters using OrthoMCL [26].

We identified 29,835 orthologous gene clusters containing 70,114 ORFs from the three organisms: i.e. 10,134, 37,382, and 22,598 ORFs longer than 100 amino acids long, respectively, from *P. bursaria*, *P. tetraurelia*, and *T. thermophila*. Figure 1 shows the number of orthologous gene clusters shared among the three organisms. Of the 29,835 orthologous gene clusters, 3,421 were common to all three organisms, 2,854 were unique to *Paramecium* species, and 2,330 were unique to *P. bursaria.*

### Differential gene expression between symbiont-bearing and symbiont-free conditions

We compared gene expressions of symbiont-bearing and symbiont-free *P. bursaria* to elucidate the genetic control for establishment of secondary symbiosis*.* Of the 10,557 transcripts, 6,698 were significantly differentially expressed between symbiont-bearing and symbiont-free cells with false discovery rates (FDR) < 0.05 (Additional file 3).

**Figure 2:**
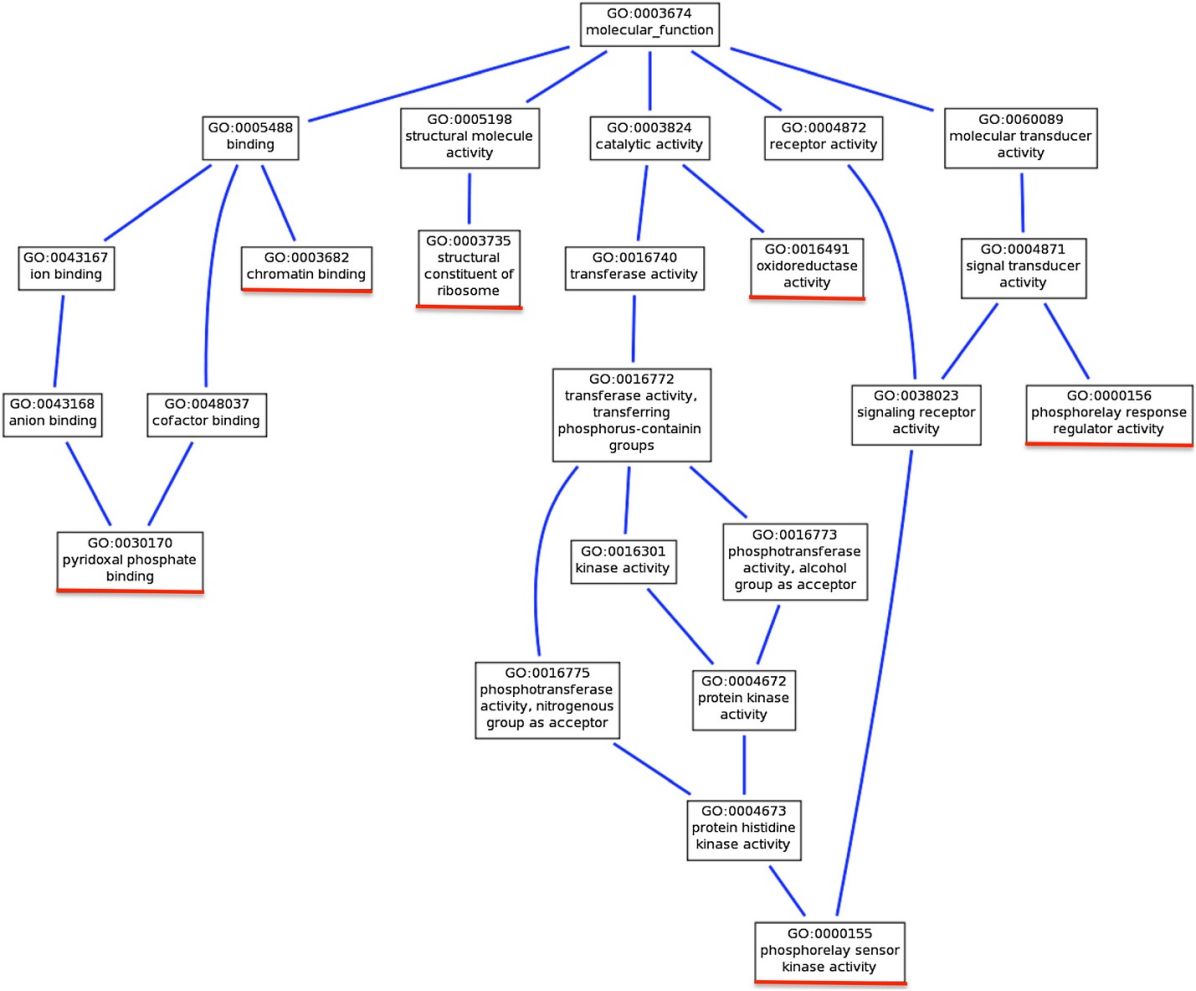
**Parent–child relation between gene ontology (GO) molecular function terms viewed by AmiGO (** http://amigo.geneontology.org/ **).** Red lines show the six highest ranking GO molecular function terms based on *P*-values in Table 1.

The positive and negative values of log2 Fold Change (logFC) show that the sequences were up-regulated and down-regulated in symbiont-bearing cells compared to symbiont-free cells. The parametric analysis of gene set enrichment (PAGE) [27] detected enrichment in the 17 gene ontology (GO) terms, i.e. 8 biological processes, 3 cellular components, and 6 molecular functions based on the logFC between symbiont-bearing and symbiont-free cells with false discovery rates (FDR) < 0.05 (Table 1). Figure 2 portrays a parent–child relation between GO molecular function terms including the six highest ranking GO terms based on PAGE *P*-values: oxidoreductase activity (*Z* = -6.879), structural constituent of ribosome (*Z* = -4.153), pyridoxal phosphate binding (*Z* = -4.015), phosphorelay response regulator activity (*Z* = 3.310), chromatin binding (*Z* = 3.107), and phosphorelay sensor kinase activity (*Z* = 2.901). A closer examination of individual proteins assigned to these GO terms revealed that trans-2-enoyl-CoA (oxidoreductase activity), aminotransferases (pyridoxal phosphate binding) and ribosomal proteins tended to be down-regulated, whereas transcriptional activator Myb-related proteins (chromatin binding), and signal transduction histidine kinase (phosphorelay response regulator activity and phosphorelay sensor kinase activity) tended to be up-regulated in symbiont-bearing cells relative to symbiont-free cells.

**Table 1:** **Enrichment of gene ontology terms in differentially expressed sequences in** ***P. bursaria*** **detected by PAGE**

GO_name	GO_id	Number of sequences	***Z*** score	***P*** -value	False discovery rate
BP oxidation–reduction process	GO:0055114	135	-6.992	2.71E-12	3.28E-10
MF oxidoreductase activity	GO:0016491	92	-6.879	6.04E-12	3.65E-10
BP metabolic process	GO:0008152	104	-5.659	1.52E-08	6.15E-07
BP carbohydrate metabolic process	GO:0005975	33	-4.656	3.23E-06	9.76E-05
CC integral to membrane	GO:0016021	196	-4.399	1.09E-05	0.0003
BP translation	GO:0006412	56	-4.187	2.83E-05	0.0006
MF structural constituent of ribosome	GO:0003735	57	-4.153	3.29E-05	0.0006
CC ribosome	GO:0005840	54	-4.044	5.25E-05	0.0008
MF pyridoxal phosphate binding	GO:0030170	13	-4.015	5.96E-05	0.0008
MF phosphorelay response regulator activity	GO:0000156	40	3.310	0.0009	0.0102
BP phosphorelay signal transduction system	GO:0000160	40	3.310	0.0009	0.0102
BP ATP hydrolysis coupled proton transport	GO:0015991	16	-3.239	0.0012	0.0121
MF chromatin binding	GO:0003682	21	3.107	0.0019	0.0176
BP DNA replication	GO:0006260	18	3.008	0.0026	0.0227
CC intracellular	GO:0005622	95	-2.953	0.0031	0.0254
MF phosphorelay sensor kinase activity	GO:0000155	26	2.901	0.0037	0.0281
BP biosynthetic process	GO:0009058	16	-2.798	0.0051	0.0366

Notes: GO, gene ontology; PAGE, parametric analysis of gene set enrichment; BP, biological process; MF, molecular function; and CC, cellular component. We used log2 Fold Change values between symbiont-bearing and symbiont-free cells to calculate *Z* scores and corresponding *P*-values for each GO term.

Based on the knowledge of *P. bursaria* accumulated to date, functions can be inferred for some of the six highest ranking GO terms. Down-regulation of ribosomal proteins in symbiont-bearing *P. bursaria* cells suggests that algal proteins with functions equivalent to those of the host *Paramecium* cells are transferred to the host through the PV membrane. Consequently transcriptional activity of the host was reduced. However, no report to date has described a demonstration showing that the proteins synthesized by the algae and transferred to the host cell through photosynthetic products, mainly maltose, are transferred from the algae [7, 9, 11].

Up-regulation of signal transduction histidine kinase in symbiont-bearing *P. bursaria* cells suggests that the histidine kinases play an important role in signal perception in this secondary symbiosis, as shown in the primary symbiosis by bacteria [28] for the adaptation and survival of various organisms to harsh environmental conditions [29]. Sensor histidine kinases are known to play important roles in several eukaryotic species including yeasts, fungi, slime molds, and higher plants [30–32]. Symbiont-bearing *P. bursaria* cells acquire various stress resistance through endosymbiosis with *Chlorella* spp [33–36].

In addition to the genes of enriched GO terms discussed above, heat shock 70 kDa protein (Hsp70) and glutathione S-transferase (GST) genes were up-regulated and down-regulated as shown by the positive and negative values of logFC, respectively, in symbiont-bearing cells compared to symbiont-free cells (Table 2). Of the 10,557 unigenes, 8 were annotated as Hsp70 with logFC of -1.3 to 5.6, with a median of 0.92. Symbiont-bearing *P. bursaria* cells are known to show a higher survival ratio against nickel chloride, high temperature, and hydrogen peroxide than the symbiont-free cells show [35, 36]. Furthermore, *P. caudatum* cells reportedly acquire heat-shock resistance by infection of endonucler symbiotic bacteria *Holospora*[37–39], and osmotic-shock resistance [40]. Hori and Fujishima [39] reported that *H. obtusa*-bearing paramecia expressed high levels of *Hsp70* mRNA even at 25°C. The up-regulation of the transcripts encoding Hsp70 might be related to the host’s tolerance to environmental fluctuations. Of the 10,557 transcripts, 7 were annotated as GST and tended to be down-regulated with logFC of -5.7 to -0.12, with a median of -0.85 (Table 2). This enzyme is related to protection of cells from oxidative stress, as shown by McCord and Fridovich [41] and by Veal et al. [42]. Although it was conceivable that photo-oxidative stress in symbiont-bearing *P. bursaria* cells is greater than that in symbiont-free ones, our data showed opposite results from the prediction. A similar result was obtained by Hörtnagl and Sommaruga [33]. They suggested that the presence of algal symbionts minimizes photo-oxidative stress [33]. Consequently, different expression levels in these genes between symbiont-free and symbiont-bearing *P. bursaria* agree well with differences in cytological phenomena observed in these paramecia, suggesting that these proteins appear to be involved in the symbiosis. Immunological detections of the gene products and comparisons of the amount of the antigens or qualitative PCR between the symbiont-free and the symbiont-bearing paramecia are necessary for future experiments. To ascertain the molecular mechanisms controlling the secondary symbiosis between *Paramecium* and *Chlorella* cells, transcriptome analyses and proteome analyses of the symbiotic *Chlorella* alone cultivated in algal culture medium and the same *Chlorella* inside the host *Paramecium* are necessary for future studies.

**Table 2:** **Transcripts encoding glutathione S-transferase and heat shock 70 kDa protein in** ***P. bursaria***

Trinity transcript name	Annotation from the SwissProt database	logFC
Heat shock 70 kDa protein		
comp43044_c0	sp|P09446|HSP7A_CAEEL|Heat shock 70 kDa protein A OS = *Caenorhabditis elegans*	5.601
comp36402_c4	sp|P09446|HSP7A_CAEEL|Heat shock 70 kDa protein A OS = *Caenorhabditis elegans*	4.183
comp36402_c6	sp|P14834|HSP70_LEIMA|Heat shock 70 kDa protein (Fragment) OS = *Leishmania major*	1.975
comp36402_c1	sp|Q9S9N1|HSP7E_ARATH|Heat shock 70 kDa protein 5 OS = *Arabidopsis thaliana*	1.555
comp37280_c1	sp|P37899|HSP70_PYRSA|Heat shock 70 kDa protein OS = *Pyrenomonas salina*	0.287
comp43771_c0	sp|P09446|HSP7A_CAEEL|Heat shock 70 kDa protein A OS = *Caenorhabditis elegans*	-0.594
comp41901_c0	sp|Q9S7C0|HSP7O_ARATH|Heat shock 70 kDa protein 14 OS = *Arabidopsis thaliana*	-1.076
comp41912_c0	sp|F4JMJ1|HSP7R_ARATH|Heat shock 70 kDa protein 17 OS = *Arabidopsis thaliana*	-1.337
Glutathione S-transferase		
comp37410_c0	sp|P78417|GSTO1_HUMAN|Glutathione S-transferase omega-1 OS = *Homo sapiens*	-0.119
comp32377_c0	sp|Q9ZRT5|GSTT1_ARATH|Glutathione S-transferase T1 OS = *Arabidopsis thaliana*	-0.288
comp36943_c0	sp|Q9ZVQ3|GSTZ1_ARATH|Glutathione S-transferase Z1 OS = *Arabidopsis thaliana*	-0.748
comp37841_c0	sp|Q9ZRT5|GSTT1_ARATH|Glutathione S-transferase T1 OS = *Arabidopsis thaliana*	-0.851
comp36483_c0	sp|Q9ZRT5|GSTT1_ARATH|Glutathione S-transferase T1 OS = *Arabidopsis thaliana*	-1.557
comp35816_c1	sp|P78417|GSTO1_HUMAN|Glutathione S-transferase omega-1 OS = *Homo sapiens*	-1.564
comp36242_c0	sp|P16413|GSTMU_CAVPO|Glutathione S-transferase B OS = *Cavia porcellus*	-5.749

## Conclusion

This study is the first whole transcriptome analysis conducted between symbiont-free and symbiont-bearing *P. bursaria*. Results showed *P. bursaria* genes that differentially expressed in symbiont-bearing and symbiont-free conditions. We showed that genes for glutathione S-transferase, trans-2-enoyl-CoA, aminotransferases, and ribosomal proteins are down-regulated, and that genes for Hsp70, transcriptional activator Myb-related proteins, and signal transduction histidine kinase are up-regulated in the symbiont-bearing *P. bursaria*. Our results enable us to understand the molecular mechanism for establishment of the secondary symbiosis and for the host evolutionary adaptation to global climate change.

## Methods

### Strains and cultures

Symbiont-free *P. bursaria* strain Yad1w was produced from *Chlorella* sp.-bearing *P. bursaria* strain Yad1g cells (syngen 3, mating type I) through repeated cloning and cultivation under dark conditions. The Yad1g cell strain was collected in Yamaguchi, Japan in 2004. Symbiont-bearing strain of Yad1g1N cells was used for symbiont-bearing cells. The Yad1g1N strain was produced by infection of cloned symbiotic *Chlorella variabilis* (formerly *C. vulgaris*) strain 1 N cells to the Yad1w cell [15]. *Paramecium* strains used for this study were provided by Symbiosis Laboratory, Yamaguchi University with support in part by the National Bio-Resource Project of the Ministry of Education, Culture, Sports, Science and Technology, Japan. The culture medium used was 1.25% (w/v) fresh lettuce juice in modified Dryl’s solution (MDS) [43] (KH_2_PO_4_ was used instead of NaH_2_PO_4_ · 2H_2_O), inoculated with a non-pathogenic strain of *Klebsiella pneumoniae* one day before use [44]. In ordinary cultures, several hundred cells were inoculated into 2 ml culture medium. Then 2 ml of fresh culture medium was added on each of the next 12 days. One day after the final feeding, the cultures were in the early stationary phase of growth. All cells used in the present experiments were at this phase. Cultivation and all experiments were performed at 25 ± 1°C. In the case of the symbiont-bearing strain of Yad1g1N, the cells were cultivated under constant light conditions: 20–30 μmol photon/m^2^/s.

### Transcriptome sequencing

Total RNA was isolated from 400,000 cells of symbiont-bearing and symbiont-free *P. bursaria* cells using Trizol reagent (Invitrogen Corp.) according to the manufacturer’s protocol. To construct three RNA-seq libraries from mRNAs of *P. bursaria,* the total RNA was isolated from three independent cultures of symbiont-free and symbiont-bearing *P. bursaria.* After suspension in Trizol reagent, the symbiont-bearing and symbiont-free cells were stirred in the presence of 600 μl of 0.5 mm zirconia/silica beads (BioSpec Products Inc.) to break cell walls of the symbiotic algae. The RNA was treated with RNase-free DNase and was cleaned up using RNeasy Mini (Qiagen Inc.) according to the manufacturer’s protocol. RNA integrity was confirmed (2100 Bioanalyzer; Agilent Technologies Inc.) with a minimum RNA integrated number value of 7.6. The samples for transcriptome analyses were prepared using Illumina’s kit following the manufacturer’s recommendations. First, mRNA was purified from 0.5 μg of total RNA of symbiont-bearing or symbiont-free cells using oligo (dT) magnetic beads. Following purification, the mRNA was fragmented into small pieces using divalent cations under elevated temperature. The cleaved RNA fragments were used for first strand cDNA synthesis using SuperScript II Reverse Transcriptase (Invitrogen Corp.) and random primers. Second strand cDNA synthesis was conducted next. These cDNA fragments then went through an end repair process and ligation of adapters. These products were purified and enriched with PCR to create the final cDNA library. Multiplex sequencing of paired-end reads was performed on an Illumina Hiseq2000 instrument at NIBB Core Research Facilities, followed by raw data processing, base-calling, and quality-control by manufacturer’s standard pipeline using RTA, OLB, and CASAVA. The output sequence quality was inspected using the FastQC program (http://www.bioinformatics.bbsrc.ac.uk/projects/fastqc/).

### *De novo*assembly

The reads were cleaned up with cutadapt program by trimming low-quality ends (<QV30) and adapter sequences, and by discarding reads shorter than 50 bp. *De novo* assembly of the clean reads was conducted using Trinity [20] in the paired-end mode with an option ‘–min_kmer_cov = 2’.

### Differential expression analysis

Data of two biological replicates were used in this analysis for each condition. Using scripts included the Trinity package suite [20], cleaned reads were aligned to the Trinity-assembled transcripts using Bowtie [45]. Then transcript abundance was estimated using RSEM [46]. We used the edgeR package [47] of Bioconductor to identify genes that are differentially expressed between the conditions. To adjust for library sizes and skewed expression of transcripts, the estimated abundance values were normalized using the Trimmed Mean of M-values (TMM) normalization method [48] included in the edgeR package. Based on a negative binomial model implemented in edgeR, genes that were differentially expressed between symbiont-bearing and symbiont-free *P. bursaria* samples were identified.

### Functional annotation of assembled contigs

Nucleotide sequences of *Chlorella variabilis* NC64A were obtained from the DOE Joint Genome Institute (JGI) site (http://genome.jgi.doe.gov/ChlNC64A_1/ChlNC64A_1.home.html). The assembled contigs that matched the *Chlorella* sequences indicated by MEGABLAST search (E-value cutoff of 1e-40) were excluded from analyses. We performed a BLASTX search (with the ciliate nuclear genetic code and E-value cutoff of 1e-5) of the *P. bursaria* transcripts against the UniProtKB Swiss-Prot and Uniref90 protein sequence databases [21] and assigned the functional annotations of the most similar protein sequences.

The protein-coding region of RNA sequences was predicted using OrfPredictor with the ciliate nuclear genetic code [23]. For sequences having BLASTX hits, the frames used by BLASTX were used for identifying the coding regions of the sequences. For sequences without BLASTX hits, the coding regions were predicted based on the intrinsic signals of the sequences. For comparative analysis, protein-coding sequences of *P. tetraurelia* were obtained from ParameciumDB (http://paramecium.cgm.cnrs-gif.fr). Those of *T. thermophila* were retrieved from Tetrahymena Genome Database (http://www.ciliate.org/). Proteins from the three organisms (*P. bursaria*, *P. tetraurelia*, and *T. thermophila*) were grouped into ortholog clusters using OrthoMCL [26] with BLASTP E-value cutoff of 1e-5 and inflation parameter of 1.5.

### Gene ontology (GO) enrichment analysis

We conducted a domain search of the *P. bursaria* transcripts against the Pfam database release 26.0 [49]. Gene ontology (GO) terms were assigned to each transcript using the pfam2go conversion table (http://www.geneontology.org/external2go/pfam2go) [50]. We performed parametric analysis of gene set enrichment or PAGE [27] to test over-representation and under-representation of GO terms based on the logFC between symbiont-bearing and symbiont-free cells.

### Availability of supporting data

The data sets supporting the results of this article are available in the DDBJ Sequence Read Archive (DRA) (accession number DRA000907, http://trace.ddbj.nig.ac.jp/DRASearch/submission?acc=DRA000907).

## Electronic supplementary material

Additional file 1: **Histogram showing the distribution of lengths for protein-coding sequences in** ***P. bursaria*** **(A),** ***P. tetraurelia*** **(B), and** ***T. thermophila*** **(C).** The *x*-axis shows the length (bp) of the sequences. The *y*-axis shows their frequencies. (PDF 372 KB)

Additional file 2: **Histogram showing the distribution of G + C contents for protein-coding sequences in** ***P. bursaria*** **(A),** ***P. tetraurelia*** **(B), and** ***T. thermophila*** **(C).** The x-axis shows the G + C content (%) of the sequences. The *y*-axis shows their frequencies. G + C content is defined as 100 × (G + C)/(A + T + G + C). (PDF 264 KB)

Additional file 3: **Data for** ***Paramecium bursaria*** **transcripts.** The first five columns show the Trinity sequence name, length (bp), and functional annotations from different databases: Swiss-Prot, UniRef90, and gene ontology (GO). The remaining four columns show data of differential expression analysis using edgeR: log2 Fold Changes (logFC), log-counts-per-million (logCPM), *P*-values, and False Discovery Rate (FDR) adjusted *P*-values. (CSV 3 MB)
