# Supplementary figures and images for: Comparison of gene expression of Paramecium bursaria with and without Chlorella variabilis symbionts
Source: BMC Genomics. 2014 Mar 10;15(1):183. doi: 10.1186/1471-2164-15-183 (PMC4029085; doi:10.1186/1471-2164-15-183)

*Paramecium bursaria*

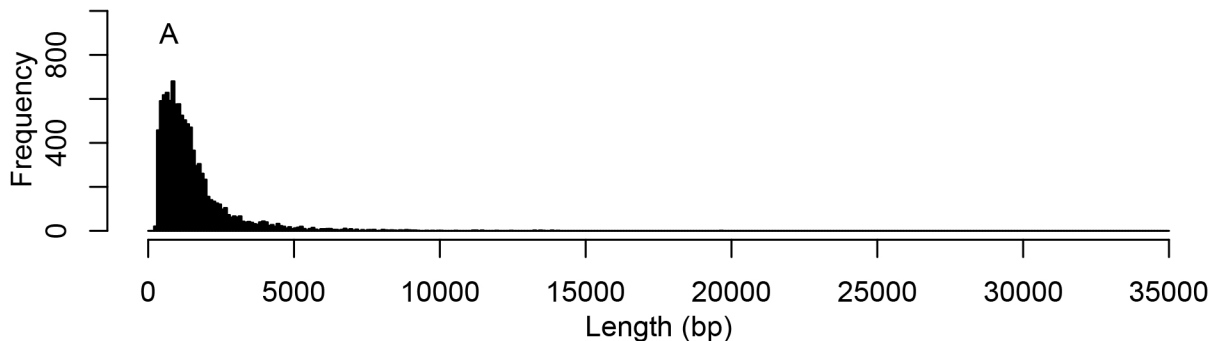

*Paramecium tetraurelia*

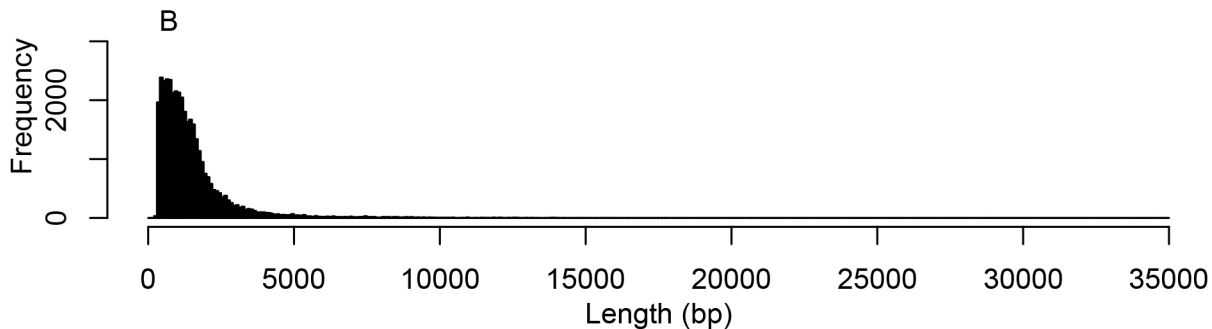

*Tetrahymena thermophila*

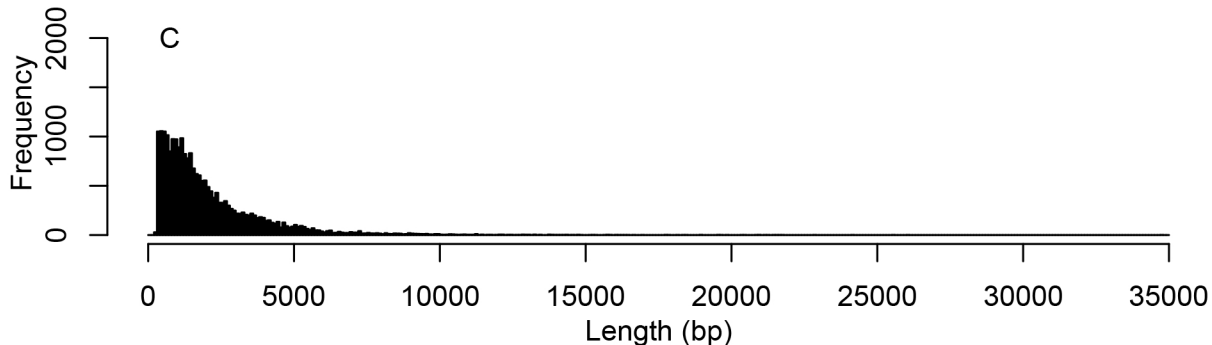

Supplement: Supplementary file 1 — Additional file 1: Histogram showing the distribution of lengths for protein-coding sequences in P. bursaria (A), P. tetraurelia (B), and T. thermophila (C). The x-axis shows the length (bp) of the sequences. The y-axis shows their frequencies. (PDF 372 KB) [file 12864_2013_7024_MOESM1_ESM.pdf]

*Paramecium bursaria*

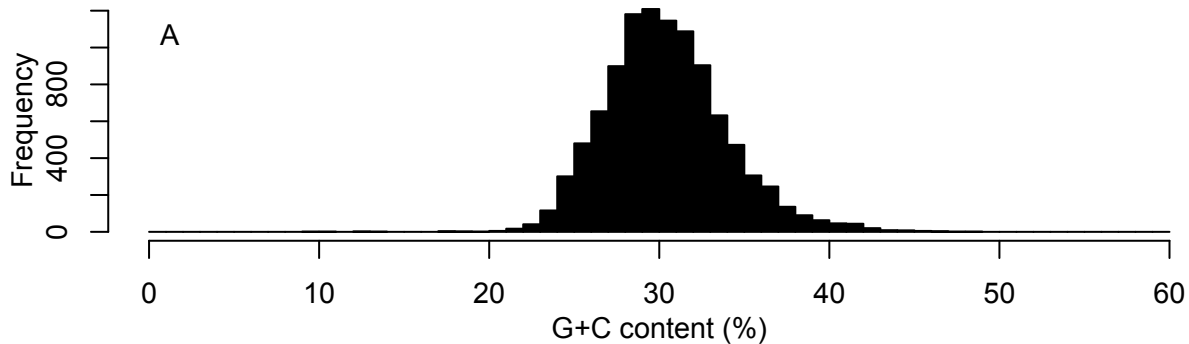

*Paramecium tetraurelia*

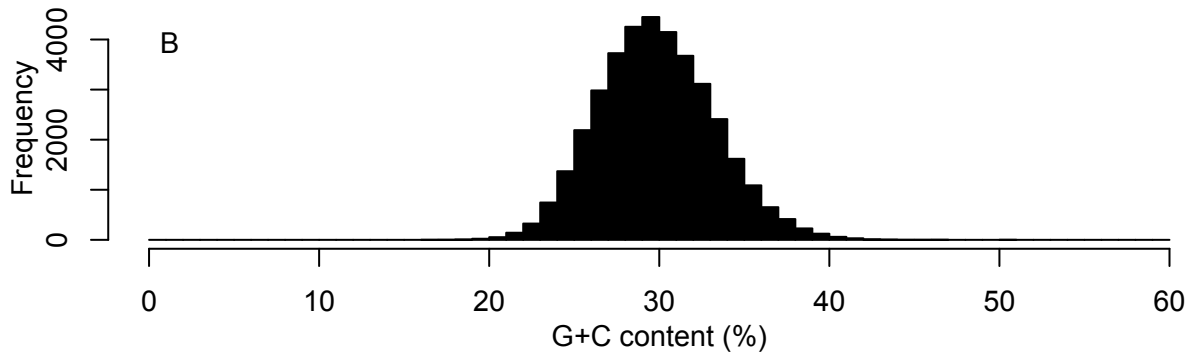

*Tetrahymena thermophila*

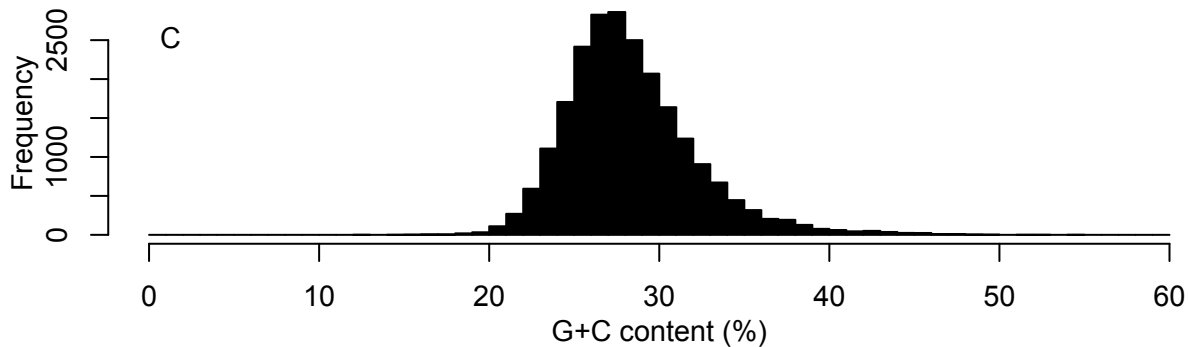

Supplement: Supplementary file 2 — Additional file 2: Histogram showing the distribution of G + C contents for protein-coding sequences in P. bursaria (A), P. tetraurelia (B), and T. thermophila (C). The x-axis shows the G + C content (%) of the sequences. The y-axis shows their frequencies. G + C content is defined as 100 × (G + C)/(A + T + G + C). (PDF 264 KB) [file 12864_2013_7024_MOESM2_ESM.pdf]
